# Corticotropin-Releasing Factor Receptor 1 in the Anterior Cingulate Cortex Mediates Maternal Absence-Induced Attenuation of Transport Response in Mouse Pups

**DOI:** 10.3389/fncel.2018.00204

**Published:** 2018-07-13

**Authors:** Sachine Yoshida, Ryuko Ohnishi, Yousuke Tsuneoka, Yuka Yamamoto-Mimura, Reiko Muramatsu, Tadafumi Kato, Hiromasa Funato, Kumi O. Kuroda

**Affiliations:** ^1^Department of Anatomy, Faculty of Medicine, Toho University, Tokyo, Japan; ^2^Precursory Research for Embryonic Science and Technology (PRESTO), Japan Science and Technology Agency (JST), Saitama, Japan; ^3^Laboratory for Affiliative Social Behavior, Center for Brain Science, RIKEN, Saitama, Japan; ^4^Department of Bioscience and Biotechnology, Faculty of Agriculture, University of the Ryukyus, Nishihara, Japan; ^5^Laboratory for Molecular Dynamics of Mental Disorders, Center for Brain Science, RIKEN, Saitama, Japan; ^6^International Institute for Integrative Sleep Medicine, University of Tsukuba, Tsukuba, Japan

**Keywords:** mouse pup, maternal absence, transport response, anterior cingulate cortex, corticotropin-releasing factor receptor 1

## Abstract

A human infant initially shows non-selective sociality, and gradually develops selective attachment toward its caregiver, manifested as “separation anxiety.” It was unclear whether such sophistication of attachment system occurs in non-human mammals. To seek a mouse model of separation anxiety, we utilized a primitive attachment behavior, the Transport Response, in that both human and mouse newborns immediately stop crying and stay immobile to cooperate with maternal carrying. We examined the mouse Transport Response in three social contexts: 30-min isolation in a novel environment, 30-min maternal absence experienced with littermates in the home cage, and the control home-cage condition with the mother and littermates. The pups after postnatal day (PND) 13 attenuated their Transport Response not only in complete isolation but also by maternal absence, and activated several brain areas including the periventricular nucleus of the hypothalamus, suggesting that 30-min maternal absence was perceived as a social stress by mouse pups after PND13. This attenuation of Transport Response by maternal absence was independent with plasma corticosterone, but was diminished by prior administration of a corticotropin-releasing factor receptor 1 (CRFR1) antagonist. Among 18 brain areas examined, only neurons in the anterior cingulate cortex (ACC) co-express *c-fos* mRNA and CRFR1 after maternal absence. Consistently, excitotoxic ACC lesions inhibited the maternal absence-induced attenuation of Transport Response. These data indicate that the expression of mouse Transport Response is influenced not only by social isolation but also by maternal absence even in their home cage with littermates after PND13, at least partly via CRF-CRFR1 signaling in the ACC.

## Introduction

Human infants are able to recognize their mother (or, more generally, primary caregiver) using visual, auditory, and/or olfactory cues even from the first month. And after 6 months of age, infants start to form a selective bond with their mother, and show signs of distress by maternal absence (separation anxiety) or by approach of unfamiliar person (stranger anxiety) (Spitz, 1965; Bowlby, 1969; Cassidy, 2008). Such fine adaptations of their behaviors to a given social context is vital for infant survival, and is acquired as an important component of the attachment system.

To understand brain mechanisms underlying the infant attachment in mammals, numerous animal studies have been trying to elucidate the neurobiological influences of maternal separation on the pup’s brain (Kuhn and Schanberg, 1998; Pryce and Feldon, 2003). However, in many rodent studies, “maternal separation” condition was not simply maternal absence but rather complete social isolation in a novel environment (Stanton et al., 1988; Plotsky and Meaney, 1993; Horii-Hayashi et al., 2013), which is actually the deprivation from three familiar figures; the mother, littermates, and home environment. Some studies investigated the effects of maternal absence on their littermates experienced in the home cage, but the duration for these “maternal absence” procedure was generally set to 24 h (termed “maternal deprivation”), because the major endocrinological changes were observed only after 4 h of maternal separation (Levine et al., 1991; Schmidt et al., 2004). To our knowledge, there is no animal study that aims to identify the brain area required for response to maternal absence for as less as 30 min, which is a physiologically normal duration for rodent pups (see below).

To seek such areas, we examined a pup’s response during the maternal transport. Several researchers described that maternal oral transport induces a characteristic compact posture in carried infants of many quadruped mammals (Eibl-Eibesfeldt, 1951; Sauer, 1967; Schaller, 1972), and this postural regulation has been studied experimentally in laboratory rats and named as “transport response” (Brewster and Leon, 1980; Wilson et al., 1984). The transport response can be easily induced by picking-up a pup, lightly pinching the skin on the back of the pup’s neck with the experimenters’ fingers to mimic the maternal oral grasping (Brewster and Leon, 1980; Wilson et al., 1984). Although previous studies described only about the postural changes during transport response, the immobility was also implicit in this response. Because the pup’s immobility enabled to carry it by maternal oral grasp or manual picking-up and to observe the postural changes. We have confirmed their findings in laboratory mouse pups, and further identified the same set of responses (immobility, reduction of heart rate and distress vocalizations) in human infants and mouse pups (Esposito et al., 2013; Yoshida et al., 2013). These calming responses observed in human infants and behavioral inhibition responses in young of non-human mammals are collectively called as the “Transport Response” hereafter.

Mouse Transport Response emerges transiently before weaning, reaching a peak in the second postnatal week and gradual attenuation from postnatal day (PND) 15, coinciding with the maturation of pups’ visual and ambulatory ability (Yoshida et al., 2013). Somatosensory and proprioceptive inputs are both required for induction, so that when the pups’ proprioception was inhibited, the pups were struggled even during maternal carrying, and retarded maternal rescue significantly. Therefore, the function of Transport Response seems to facilitate maternal carrying (Esposito et al., 2013). Taken together, the Transport Response can be regarded as a primitive attachment behavior to maintain maternal proximity.

In this study, we focused on the mouse Transport Response as a representative attachment behavior to examine whether its expression changed after 30 min maternal absence. We first showed that, after PND13, the Transport Response was attenuated by maternal absence even when they stayed together with littermates in their home cage. And through the brain-wide activation mapping analysis, we infer the importance of the CRF-CRFR1 signaling in the anterior cingulate cortex (ACC) for the social context-dependent Transport Response regulation.

## Materials and Methods

### Animals

All animal experiments were approved by animal experiment committee of RIKEN and Toho University (Approved protocol ID #17-41-349). C57BL/6 mice were purchased from Japan SLC (Shizuoka, Japan) and CLEA Japan (Tokyo, Japan). Mice were maintained under a 12 h light/dark cycle (lights-on 08:00) with food and water *ad libitum*. C57BL/6 pups were culled to six, consisting of both males and females until PND3. Each pup received an injection of animal tattoo ink (Natsume, Japan) into the forepaw or the footpad to distinguish them from one another. All experiments were performed between 10:00 and 12:00.

### Measurement of Transport Response

To induce Transport Response, the dorsal skin of the pup’s neck was pinched using the experimenters’ fingers with just enough force to pick up the pup but not so much as to leave a mark on the skin after release as previously described (Esposito et al., 2013; Yoshida et al., 2013). The amount of pinched skin was also minimal, so that the skin around the neck was never tightly squeezed to inhibit the body movement of the pup. We used powder-free latex gloves (Diamond Grip, Microflex, Reno, NV, United States) for manual carrying according to the animal safety regulations of RIKEN and Toho University and to increase friction at the finger tips. Each pup was picked up only once to examine the Transport Response from PND8 to PND20. The mice were manually picked up for 15 s or until they started struggling in the air (i.e., the rapid and anti-gravitational movement throughout the body). Behavioral tests were recorded with a Handycam HDR-SR12 video camera (Sony, Japan). The movie replay and editing were conducted using the Picture Motion Browser (Sony) and Premiere Pro CS4 (Adobe, CA, United States) software programs. The immobilization times were manually measured using stopwatches for the latency of the initial struggling from pick-up. Video analyses were performed by at least two raters without any prior information about the experimental manipulations.

### Modulation of Social Conditions

The pups were exposed to one of three social conditions and then picked up manually to examine the immobilization time: (1) a pup was isolated in a novel cup (125 mm φ × 85 mm) with the new bedding for 30 min (ISO); (2) after quick and gentle removal of the mother from the home cage without touching pups, pups were exposed to 30 min maternal absence in the home cage (MAB); and (3) mother and pups were kept undisturbed until the experiment (CTL). The experimental pups were decapitated to collect blood or perfused right after the 30 min experimental period for histological analyses of the brain.

### Preparation of Brain Sections for Histological Analyses

The pups were deeply anesthetized with sodium pentobarbital (50 mg/kg, intraperitoneally), then perfused transcardially with 4% (w/v) paraformaldehyde (PFA) in phosphate-buffered saline (PBS, pH 7.4). The brains were removed, immersed in the same fixative at 4°C overnight, followed by cryoprotection in a series of 30% (w/v) sucrose in PBS for 1 day, embedded in O.C.T. Compound (Sakura Finetek, Japan), and stored at -20°C until cryosection. Brains were cryosectioned coronally at a thickness of 12 μm with reference to the mouse brain atlas (Paxinos and Franklin, 2013) and mounted on MAS-coated glass slides (Matsunami, Osaka, Japan). Every fifth section from the serial sections from the ACC to the locus coeruleus was processed for *in situ* hybridization (ISH) and/or immunohistochemistry (IHC).

### *In Situ* Hybridization and Immunohistochemistry

For production of non-isotopic riboprobes, total RNA of mouse brain was extracted, and cDNA was synthesized using PrimeScript RT mix (Takara, Shiga, Japan). To generate *c-fos* antisense riboprobe, cDNA mixture was amplified by PCR using primers for *c-fos* (NM_010234), forward 5×′-AGAATCCGAAGGGAACGG-3′ and reverse 5′-AATTAACCCTCACTAAAGGGGGAGGCCAGATGTGGATG-3′. The reverse primer contained an artificially introduced T3 promoter at its 5′ end (5′-AATTAACCCTCACTAAAGGG-3′) as described (David and Wedlich, 2001). The antisense probes were transcribed by T3 RNA polymerase (P2083; Promega, Madison, WI, United States) in the presence of digoxigenin-labeled UTP (Dig labeling mix; Roche Diagnostics, Switzerland) followed by precipitation with LiCl with ethanol. The brain sections were processed for ISH following a standard procedure as described (Strähle et al., 1994) with some modifications with subsequent immunohistochemical detection of CRFR1. Briefly, the sections were washed with PBS containing 0.1% Tween-20 (PBT) and immersed in proteinase K (1 μg/ml in PBT) for 2 min at 37°C. Then, they were postfixed with 4% PFA in PBS for 15 min at room temperature. The hybridization solution contained 50% of deionized formamide, 5× standard saline citrate (SSC, pH 7.0), 5 mM ethylene-diaminetetraacetic acid (pH 8.0), 0.2 mg/ml of yeast tRNA, 0.2% Tween-20, 0.2% sodium dodecyl sulfate, 10% dextran sulfate, and 0.1 mg/ml of heparin. The sections were prehybridized at 58°C in a mixture of the hybridization solution and PBT (1:1) for 30 min, immersed in the hybridization solution for 30 min, then hybridized with the riboprobes (1.5 μg/ml) at 58°C for 16 h. After hybridization, the sections were washed twice with 2× SSC containing 50% deionized formamide at 58°C for 10 min, incubated with RNase A solution (20 μg/ml) at 37°C for 30 min, rinsed twice in 2× SSC and 0.2 × SSC at 37°C (10 min each), and incubated in an alkaline phosphatase-conjugated anti-digoxigenin antiserum (1:1,000; Roche) for 2 h. Then the sections were stained blue-purple with BCIP/NBT for bright-field observations. For IHC, the sections were incubated with CRFR1 antibody (0.5 μg/ml) (Abcam, United States) and stained brown using anti-goat biotin-conjugated antibody (1:1,000), ABC peroxidase (Vector laboratories, CA, United States), and DAB solution (Nacalai tesque, Kyoto, Japan).

### Histological Analysis

Serial PND16 brain sections processed for *c-fos* mRNA ISH except the olfactory bulb were first qualitatively observed using microscopes with light condensers (Leica microsystems, Wetzlar, Germany and Olympus Corporation, Tokyo, Japan). In PND10 brain sections for ISH, photomicrographs were taken using a digital slide scanner (NanoZoomer Digital Pathology; Hamamatsu Photonics, Japan) and a microscope (Olympus). Anatomical structures were identified according to the mouse brain atlas (Paxinos and Franklin, 2013) and the previous study (Vogt and Paxinos, 2014). The number of *c-fos* mRNA expressing neurons within a 400 μm square placed in the brain areas listed in **Figure 2**, except the PFI, were counted bilaterally using ImageJ software^1^ (developed by Wayne Rasband, National Institutes of Health, Bethesda, MD) and photoshop (Adobe, CA, United States), and corrected manually. In the PFI, *c-fos* expressing neurons were observed in highly dense manner so that we quantified the signal intensity in a 400 μm square instead of using cell counts. *c-fos* mRNA expressing neurons were quantified bilaterally for each brain area of interest. For IHC analyses, serial brain sections stained with a CRFR1 antibody were observed with a bright field microscope (Olympus). A 400 μm square was placed in the middle of ACC and immuno-positive areas within the square were quantified using the ImageJ. For combination of ISH and IHC, *c-fos* mRNA expressing and CRFR1 immuno-positive neurons in the ACC were counted in a 150 μm square, first automatically with the ImageJ, and corrected manually.

### Stereotaxic Surgery Using Excitotoxic Drugs

PND14 pups were deeply anesthetized using isoflurane (1–1.2% in air via a facemask), placed in a stereotaxic frame (Narishige, Tokyo, Japan) and then the excitotoxic amino acid, N-methyl-D-aspartic acid (NMDA) (25 mg/ml, Sigma-Aldrich, St. Louis, MO, United States), was injected at the stereotaxic coordinates A 1.2 mm, L 0.1 mm, V 1 mm into both hemispheres through a glass capillary (tip diameter: 30–50 μm) by liquid pressure. Sham-operated pups were injected with saline. Stereotaxic coordinates based on the bregma zero reference point were taken from the mouse brain atlas (Paxinos and Franklin, 2013). The injected volumes were approximately 80 nl to destroy the area where *c-fos* mRNA expression was increased after MAB and ISO exposures. At PND16, pups were exposed to one of the conditions of ISO, MAB, or CTL and then picked up manually to examine Transport Response. To confirm the lesioned area, brains were removed for Nissl staining. The lesioned area in this study corresponded to the ACC (Vogt and Paxinos, 2014) of approximately 960 μm long described as A24a and A24b from Figures 19 to 27 in the brain atlas (Paxinos and Franklin, 2013).

### Pharmacological Treatment

Some PND16 pups received an intraperitoneal injection of 60 mg/kg CP-154526, non-peptide CRF1 receptor antagonist (Santa Cruz, CA, United States) 30 min prior to MAB or ISO exposure. CP-154526 were dissolved in 3% cremophor EL (SIGMA)/saline. To minimize injection stress, the mother was gently separated from the huddling pups, and then the pups were gently transferred to a small cup covered with home-cag bedding. Two experimenters injected drugs into pups at the same time to minimize the injection time-lag among the pups. After MAB or ISO exposure, the pups were picked up manually and the immobilization time was measured. At the end of the Transport Response, the pups injected CP-154526 started struggling similar to control pups. We did not measure the ultrasonic vocalizations (USVs) in PND16 pups because USV emissions during social isolation are very few even without CP-154526 (Roubertoux et al., 1996). After the video recording of Transport Response, we decapitated and perfused the pups for plasma collection and for histological analyses, respectively. The plasma was collected and stored at -20°C until use. The plasma CORT level was measured using ELISA kit AssayMax (Assaypro, St. Charles, MO, United States).

In our study, pre-injection of CP-154526 was able to inhibit the ACC activation and disinhibit ACC-dependent attenuation of Transport Response upon social isolation, but did not inhibit CORT elevation after social isolation. This is in agreement with the previous report showing that known selective CRFR1 antagonists, including CP-154526 (Gozzi et al., 2013) and MTIP (Gehlert et al., 2007) do not inhibit the increase of plasma CORT after physical stress, while they suppress the behavioral responses to stress. The reason for this phenomenon has not been fully clarified, but presumably the intraperitoneal injection of the antagonist prior to the behavioral experiment causes the CORT elevation before the antagonist starts functioning, and masks the effect on CORT by the successive behavioral experiment.

### Statistical Analysis

Statistical analysis was performed using Welch’s *t*-test or Welch’s analysis of variance (ANOVA) as appropriate, with significance set at *p* < 0.05 after *p-*value correction by Holm’s method. All statistical analyses were conducted using R version 3.3.2 (R Development Core Team). Quantitative data were presented as the mean ± SEM.

## Results

### Under Maternal Absence, Mouse Pups Attenuate Transport Response After PND13

**FIGURE 1:**
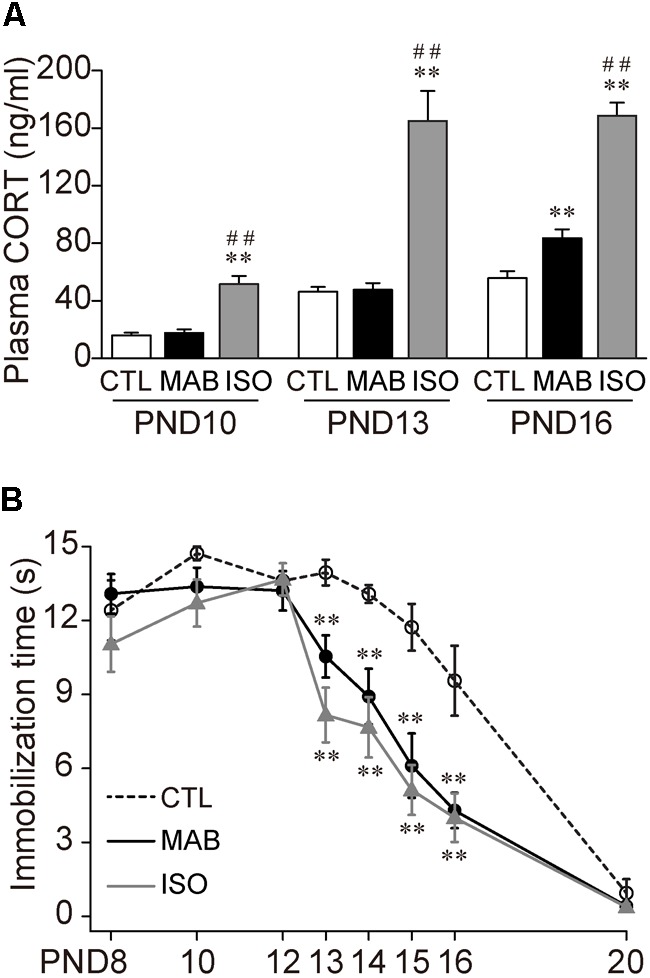
Maternal absence attenuates Transport Response with age. **(A)** Comparison of plasma CORT level after control home-cage condition with mother and littermates (CTL), maternal absence in a home-cage with littermates (MAB) and maternal absence under isolated condition in a novel environment (ISO). **(B)** Ontogenic reduction of immobilization time after CTL, MAB or ISO. ^∗∗^*p* < 0.01 vs. CTL, ^##^*p* < 0.01 vs. MAB. *n* = 12 in each group at each PND. Mean ± SEM.

First, to examine how social context regulates the expression of Transport Response, we designed three distinct social conditions: (1) 30 min complete isolation of a pup in a novel environment (ISO), (2) 30 min maternal absence experienced with littermates in their home-cage (MAB), or (3) the control home-cage condition staying with the mother and littermates (CTL). It should be noted that, in a natural environment, a mouse pup stays in the nest together with its littermates until the second postnatal week, then it gradually achieves visual and ambulatory ability by the time of weaning at 3 weeks of age. The mouse mothers stay with their litter most of the time during the light period, and periodically go out for foraging during the dark period, because they are nocturnal. In our laboratory condition, the maximal maternal absence for C57BL/6 litter was occurred in the beginning of the dark phase, and about 30 min at PND 7, and an hour at PND 14 (Ohmura et al., manuscript in preparation), comparable to the previous study in laboratory rats (Grota and Ader, 1974) and guinea pigs (Hennessy and Sharp, 1990). Therefore, the MAB condition should be common and not too stressful for mouse pups, compared with the ISO condition, which may signal an emergency situation. Consistent with this notion, the concentration of plasma CORT, the major stress hormone, increased more than twofold after the exposure of ISO than CTL condition at all age groups tested (**Figure 1A**) [PND10, ANOVA, *F*_(2,19.94)_ = 17.74, *p* < 0.0001; Pairwise comparisons, *p* = 0.55 in CTL vs. MAB, *p* = 0.0001 in ISO vs. CTL or MAB; PND13, ANOVA, *F*_(2,23.89)_ = 15.46, *p* < 0.0001; Pairwise comparisons, *p* = 0.82 in CTL vs. MAB, *p* < 0.001 in ISO vs. CTL or MAB]. On the other hand, the CORT level after MAB conditions was significantly higher than the CTL group only at PND16 [PND16, ANOVA, *F*_(2,23.25)_ = 60.07, *p* < 0.0001; Pairwise comparisons, *p* < 0.01 in CTL vs. MAB, *p* < 0.0001 in CTL vs. ISO, *p* < 0.0001 in MAB vs. ISO].

Using a different set of mouse pups, after the 30 min exposure to any of the three social conditions, the experimenter picked up each pup manually to induce the Transport Response and measured the immobilization time. There was no significant difference in the length of the immobilization time among the three groups from PND8 to PND12, and at PND20 when the Transport Response diminished (**Figure 1B**). During PND13–16, on the other hand, the immobilization time became significantly shorter in ISO than in CTL, suggesting the isolation stress reduced the Transport Response. Notably, MAB also significantly reduced the immobilization time throughout PND 13–16 [PND13, ANOVA, *F*_(2,43.14)_ = 13.74, *p* < 0.0001; Pairwise comparisons, *p* < 0.01 in CTL vs. MAB, *p* < 0.001 in CTL vs. ISO, *p* = 0.098 in MAB vs. ISO; PND14, ANOVA, *F*_(2,36.15)_ = 13.71, *p* < 0.0001; Pairwise comparisons, *p* < 0.01 in CTL vs. MAB, *p* < 0.001 in CTL vs. ISO, *p* = 0.46 in MAB vs. ISO; PND15, ANOVA, *F*_(2,36.3)_ = 12.52, *p* < 0.01; Pairwise comparisons, *p* < 0.01 in CTL vs. MAB, *p* < 0.01 in CTL vs. ISO, *p* = 0.56 in MAB vs. ISO; PND16, *F*_(2,33.02)_ = 6.03, *p* < 0.01; Pairwise comparisons, *p* < 0.01 in CTL vs. MAB, *p* < 0.01 in CTL vs. ISO, *p* = 0.81 in MAB vs. ISO].

These results indicate that maternal absence attenuates pup’s Transport Response after PND13. Also, this attenuation induced by maternal absence was not correlated with the plasma CORT concentration.

### Identification of Brain Regions Activated by Maternal Absence

**FIGURE 2:**
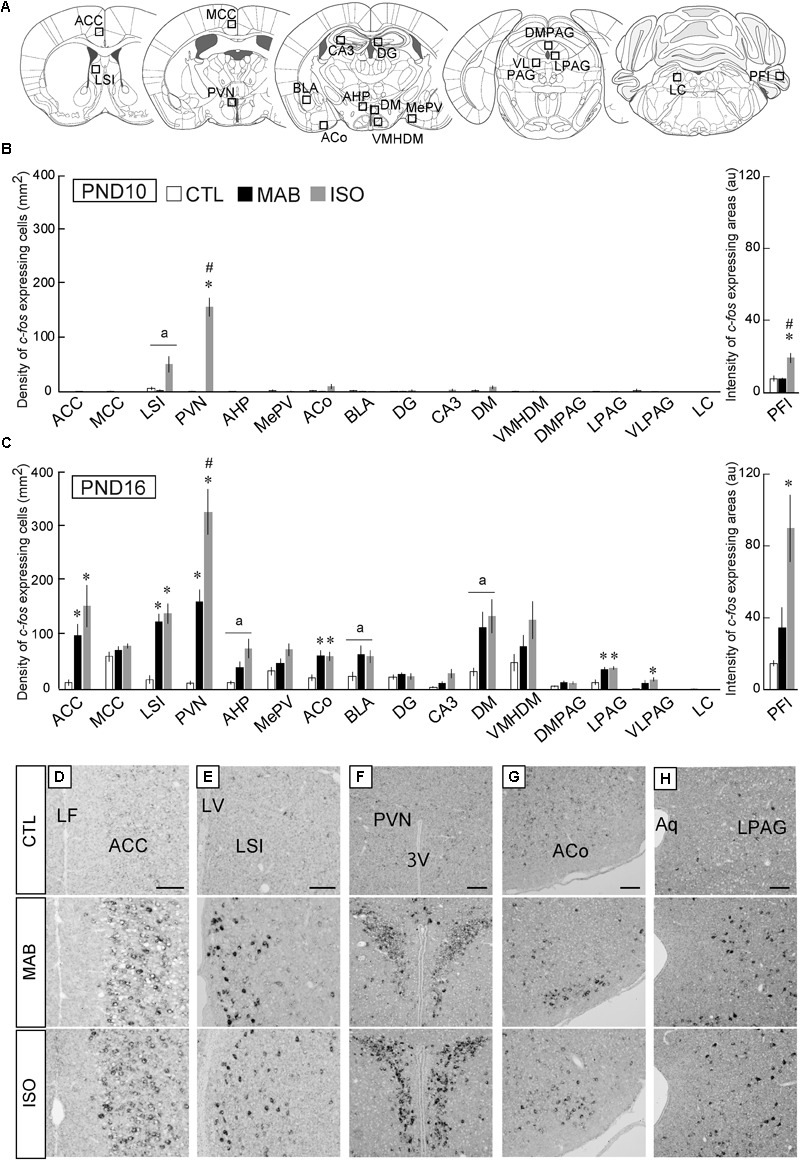
Quantitative analyses of *c-fos* mRNA expression. **(A)** Squares showed approximate positions for *c-fos* expression analyses. **(B,C)** The number of *c-fos* mRNA expressing neurons was counted in 17 brain areas including the ACC, midcingulate cortex (MCC), lateral septal nucleus intermediate part (LSI), paraventricular hypothalamic nucleus (PVN), anterior hypothalamic area posterior part (AHP), medial amygdaloid nucleus posteroventral part (MePV), anterior cortical amygdaloid nucleus (ACo), basolateral amygdaloid nucleus anterior part (BLA), dentate gyrus (DG), field CA3 of the hippocampus (CA3), dorsomedial hypothalamic nucleus (DM), hypothalamic nucleus dorsomedial part (VMHDM), dorsomedial periaqueductal gray (DMPAG), lateral periaqueductal gray (LPAG), ventrolateral periaqueductal gray (VLPAG), and the locus coeruleus (LC). In the paraflocculus (PFl), the signal intensities were measured (arbitrary unit). After CTL, MAB, or ISO, the representative *c-fos* expression patterns detected as black dots in the ACC **(D)**, LSI **(E)**, PVN **(F)**, ACo **(G)**, and LPAG **(H)** were listed. *n* ≥ 5 in each group at each PND. ^∗^*p* < 0.05 vs. CTL, ^#^*p* < 0.05 vs. MAB. (a) indicated nuclei that showed a significant difference in ANOVA, but not in a *post hoc* test. au, arbitrary unit, Mean ± SEM. Scale bars = 100 μm. Aq, aqueduct; cc, corpus callosum; LF, longitudinal fissure; LV, lateral ventricle; 3V, 3rd ventricle.

In order to identify the brain area responsible for this maternal absence-induced attenuation of Transport Response, we next performed whole brain screening for *c-fos* mRNA expression induced by CTL, MAB, and ISO, respectively. *c-fos* mRNA expression levels have been utilized as a neuronal activation marker (Chan et al., 1993). We first performed preliminary screening of *c-fos* mRNA expression in the whole brain, and selected several brain areas that showed high *c-fos* mRNA expression after MAB and/or ISO for the quantitative analysis. We also included several brain areas that have been implicated in anxiety, immobilization and responses to maternal separation, such as the medial amygdala and lateral periaqueductal gray (LPAG) (Levine et al., 1991; Wiedenmayer and Barr, 2001; Muigg et al., 2009). A total of 17 brain areas were selected for quantitative analyses: the anterior cingulate cortex (ACC), midcingulate cortex (MCC), lateral septal nucleus intermediate part (LSI), paraventricular hypothalamic nucleus (PVN), anterior hypothalamic area posterior part (AHP), medial amygdaloid nucleus posteroventral part (MePV), anterior cortical amygdaloid nucleus (ACo), basolateral amygdaloid nucleus anterior part (BLA), dentate gyrus (DG), field CA3 of the hippocampus (CA3), dorsomedial hypothalamic nucleus (DM), ventromedial hypothalamic nucleus dorsomedial part (VMHDM), dorsomedial periaqueductal gray (DMPAG), LPAG, ventrolateral periaqueductal gray (VLPAG), locus coeruleus (LC), and the paraflocculus (PFl) (**Figure 2A**^2^). Then we quantified the expression of *c-fos* mRNA in each brain area after the 30 min exposure to CTL, MAB or ISO in PND10 and PND16 pups (**Figures 2B,C**).

In PND10 pups, ISO but not MAB caused significant increase of *c-fos* expression in the PVN [ANOVA was not applicable (NA) because of very low expression in CTL and MAB samples; Pairwise comparisons, *p* = 0.36 in CTL vs. MAB, *p* < 0.001 in CTL vs. ISO, *p* < 0.001 in MAB vs. ISO], and in the PFl [*F*_(2,8.65)_ = 8.35, *p* = 0.01; Pairwise comparisons, *p* = 0.88 in CTL vs. MAB, *p* < 0.05 in CTL vs. ISO, *p* < 0.05 in MAB vs. ISO; **Figure 2B**].

On the other hand, many brain areas at PND16 showed an increase of *c-fos* expression not only by ISO but also by MAB (**Figure 2C**). The *c-fos* induction in the PVN at PND16 was largest by ISO, then MAB, and lowest by CTL (**Figures 2C,F**; *F*_(2,5.55)_ = 43.89, *p* < 0.001; Pairwise comparisons, *p* < 0.01 in CTL vs. MAB, *p* < 0.01 in CTL vs. ISO, *p* < 0.05 in MAB vs. ISO]. Therefore, the *c-fos* in the PVN was coincided well with the plasma CORT level at both PND10 and PND16, consistent with the established role of PVN governing the hypothalamic-pituitary-adrenal (HPA) axis (Schmidt et al., 2003). The *c-fos* expression in the PFl at PND16 was similar to the PVN, although the difference between CTL vs. MAB did not reach to the statistical significance [*F*_(2,5.48)_ = 8.5, *p* < 0.05; Pairwise comparisons, *p* = 0.15 in CTL vs. MAB, *p* < 0.05 in CTL vs. ISO, *p* = 0.083 in MAB vs. ISO]. The PFl has been known to regulate head-eye coordination, and a report showed that the PFI expressed *c-fos* mRNA after physical stress exposure such as light and noise (Campeau et al., 1997).

The *c-fos* expression of the ACC, on the other hand, coincided with the immobilization time rather than with the plasma CORT level; not activated by any social conditions at PND10 (NA), while activated equally by ISO and MAB at PND16 (**Figures 2C,D**; *F*_(2,5.94)_ = 12.86, *p* < 0.01; Pairwise comparisons, *p* < 0.05 in CTL vs. MAB, *p* < 0.05 in CTL vs. ISO, *p* = 0.26 in MAB vs. ISO]. The MCC, which is posterior to the ACC in the cingulate cortex, did not show any significant activation by social context (PND10, NA), [PND16, *F*_(2,7.29)_ = 2.01, *p* = 0.20; Pairwise comparisons, *p* = 0.66 in CTL vs. MAB, *p* = 0.27 in CTL vs. ISO, *p* = 0.66 in MAB vs. ISO]. The same pattern was observed in the ACo (**Figures 2C,G**) and the LPAG (**Figures 2C,H**; PND10, NA) [PND16, ACo: *F*_(2,7.53)_ = 9.6, *p* < 0.01; Pairwise comparisons, *p* < 0.05 in CTL vs. MAB, *p* < 0.05 in CTL vs. ISO, *p* = 0.96 in MAB vs. ISO; PND16, LPAG: *F*_(2,7.76)_ = 8.84, *p* < 0.05; Pairwise comparisons, *p* < 0.05 in CTL vs. MAB, *p* < 0.05 in CTL vs. ISO, *p* = 0.68 in MAB vs. ISO].

The *c-fos* expression pattern in the LSI at PND16 also showed a similar trend at PND16 [*F*_(2,6.95)_ = 29.69, *p* < 0.001; Pairwise comparisons, *p* < 0.01 in CTL vs. MAB, *p* < 0.01 in CTL vs. ISO, *p* = 0.53 in MAB vs. ISO], but less at PND10 [*F*_(2,8.91)_ = 4.94, *p* < 0.05; Pairwise comparisons, *p* = 0.33 in CTL vs. MAB, *p* = 0.067 in CTL vs. ISO, *p* = 0.067 in MAB vs. ISO; **Figures 2B,C,E**].

Thus, among 17 brain areas analyzed, the ACC, LSI, ACo, and LPAG were of special interest as possible mediators of maternal absence-induced attenuation of the Transport Response.

### CRFR1 Signaling Involves in Attenuation of Transport Response Induced by Maternal Absence and Social Isolation

The above results indicated that the attenuation of Transport Response by maternal absence was not mediated by plasma CORT. An alternative candidate mediator of this mechanism is the Corticotropin Releasing Factor (CRF, also called Corticotropin Releasing Hormone, CRH), a key stimulator of the HPA axis as well as sympathetic responses to stress. Moreover, centrally released CRF also contributes directly to anxiety-related behaviors independently of its effects on the pituitary and sympathetic systems (Henckens et al., 2016). This anxiogenic effects of CRF have been attributed to the activation of corticotropin-releasing factor receptor 1 (CRFR1), as evidenced by CRFR1 blockade or genetic targeting studies (Timpl et al., 1998), while the role of CRFR2 might be recovery from stress.

**FIGURE 3:**
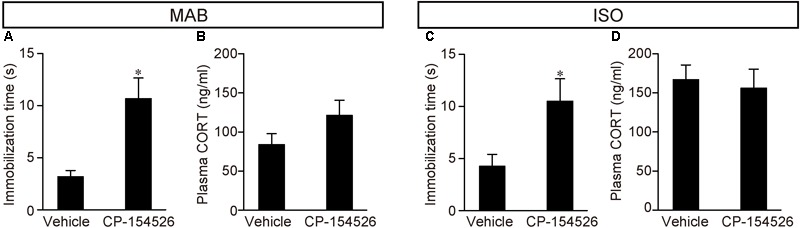
Disinhibition of Transport Response after blockade of CRFR1 signaling. Pups received pre-injection of vehicle or CP-154526 before maternal absence. After MAB **(A,B)** or ISO **(C,D)** exposure, the immobilization time and plasma CORT were compared between vehicle- and CP-154526-injected pups. ^∗^*p* < 0.05, ^∗∗^*p* < 0.01. Mean ± SEM.

Therefore, we examined whether CRFR1 signal blockade disinhibited the attenuation of Transport Response after maternal absence and/or social isolation (**Figure 3**). PND16 pups received pre-injection of CP-154526, a CRFR1 antagonist penetrating the blood-brain-barrier (Schulz et al., 1996), and then exposed to MAB or ISO. The immobilization time was longer in the pups that received CP-154526 injection than in vehicle injection, and was comparable to the immobilization time of control pups stayed with the mother in **Figure 1** (MAB in **Figure 3A**; *t* = -3.63, *df* = 5.92, *p* < 0.05, *n* = 6 each group; ISO in **Figure 3C**; *t* = -2.57, *df* = 10.69, *p* < 0.05, *n* = 8 each group). These results indicated that both maternal absence and social isolation attenuated the immobilization time during Transport Response via CRF-CRFR1 signaling.

Consistent to the findings in the **Figure 1A**, pups experienced either MAB or ISO and vehicle injection showed heightened CORT concentrations, and the plasma CORT did not significantly decrease from the injection of CP-154526 (see section “Discussion”) (MAB in **Figure 3B**; *t* = -1.57, *df* = 9.16, *p* = 0.15, *n* = 6 each group; ISO in **Figure 3D**; *t* = 0.35, *df* = 13.77, *p* = 0.73, *n* = 8 each group). The discrepant responses of immobilization time and plasma CORT confirmed their independent regulation.

### CRFR1 Expressing Neurons in the ACC Are Activated by Maternal Absence and by Isolation

To identify the action site of CRF-CRFR1 signaling during maternal absence, we then performed double detection of *c-fos* mRNA expression and CRFR1 immunoreactivity in the candidate brain areas, the ACC, LSI, ACo, and LPAG at PND16 as suggested in the ISH analyses (**Figure 2**). In addition to these areas, we also examined the central nucleus of the amygdala (CeA), which is known for high CRFR1 expression (Henckens et al., 2016) and the PVN, in which CRFR1 mRNA expression is known to be induced by severe physical stress or acute osmotic stress (Luo et al., 1994).

**FIGURE 4:**
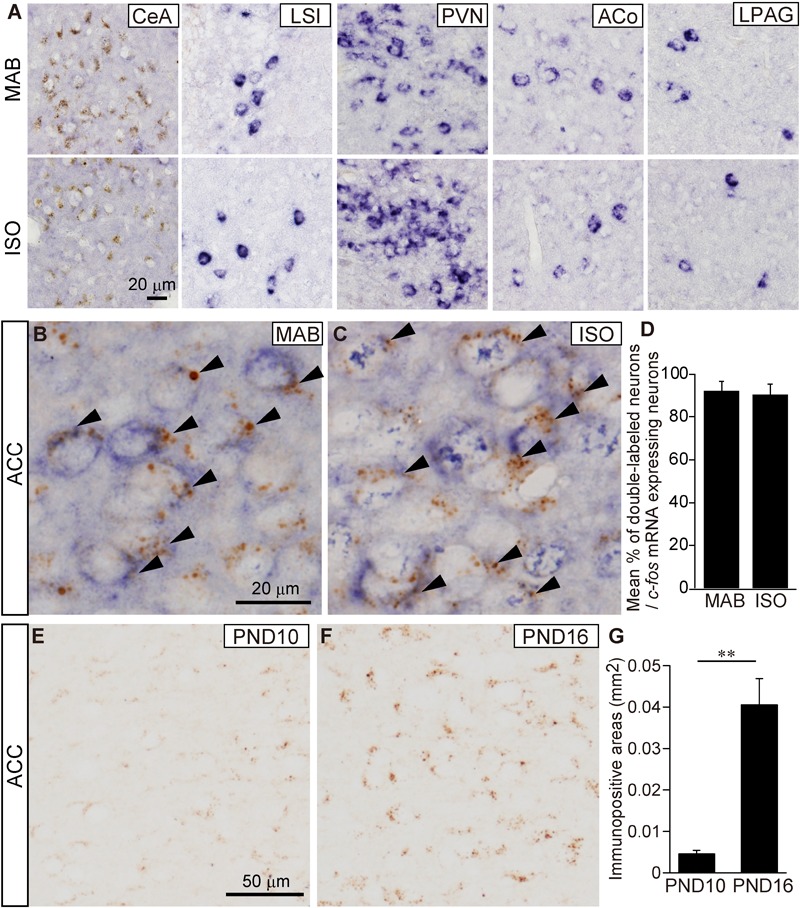
Histological characterization of *c-fos* expressing neurons in the ACC. **(A)** Representative images of *c-fos* mRNA expressing neurons (blue) and CRFR1 immuno-positive neurons (brown) in the CeA, LSI, PVN, ACo, and LPAG at PND16 after MAB or ISO exposure. Detection of *c-fos* mRNA expressing neurons and CRFR1 immuno-positive neurons in the ACC of PND16 pups exposed MAB **(B)** or ISO **(C)**. Arrowheads showed double-labeled neurons. Double-labeled neurons were counted **(D)**. Comparison of CRFR1 immuno-positive areas **(G)** in the ACC between PND10 **(E)** and PND16 **(F)**. ^∗∗^*p* < 0.01. Mean ± SEM.

As expected from our preparatory analysis, the CeA showed the abundant immunoreaction for CRFR1, but almost no *c-fos* expression by MAB or ISO (**Figure 4A**). The LSI, PVN, ACo, and LPAG exhibited clear *c-fos* expression but almost no CRFR1 immunoreaction, and hence the double labeled cells were undetectable (**Figure 4A**; *t*-test was not applicable because of almost no CRFR1 immunoreaction). In contrast, 90.7 ± 4.64% in MAB (*n* = 4) and 92.31 ± 4.35% in ISO (*n* = 4) of *c-fos* expressing neurons were also immuno-positive for CRFR1 in the ACC obtained from pups exposed to MAB and ISO (**Figures 4B–D**). Therefore, the ACC was supposed to be the best candidate regulator for the inhibition of Transport Response during maternal absence.

Moreover, during the course of the study, we found that the immunoreaction of CRFR1 at the ACC neurons increased sharply between PND10 and PND16 (**Figures 4E–G**; *t* = -5.63, *df* = 4.15, *p* < 0.01, *n* = 5, each PND), consistent to the time-course of inhibitory regulation of Transport Response by social context.

### CRFR1 Signaling Blockade Reduced the Neuronal Activation in the ACC During Maternal Absence or Isolation

To further elucidate the neural pathway responsible for the social context-dependent Transport Response regulation, we investigated the *c-fos* mRNA expression with or without CRFR1 signaling blockade by CP-154526 pre-injection in six brain areas, the PVN, ACC, LSI, ACo, LPAG, and CeA again.

**FIGURE 5:**
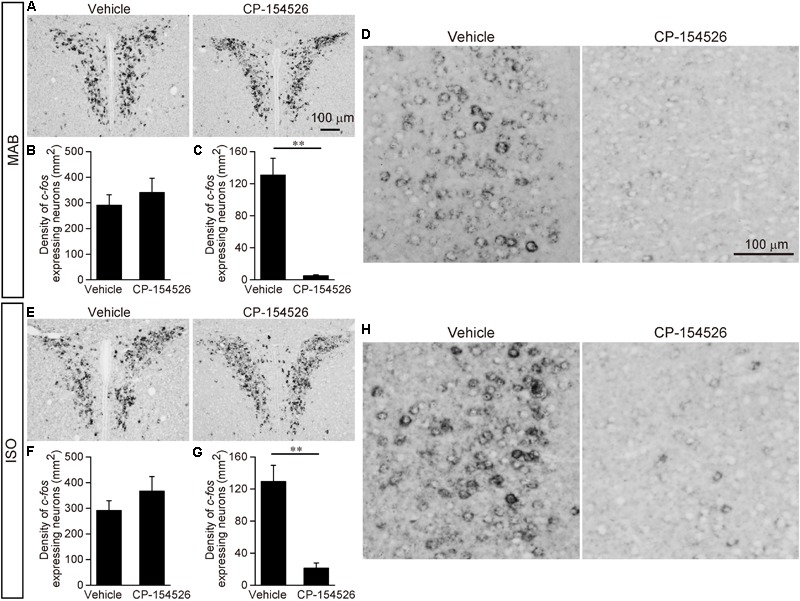
Decrease of *c-fos* expression after blockade of CRFR1 signaling. Pups received pre-injection of vehicle or CP-154526 before maternal absence. After MAB **(A–D)** or ISO **(E–H)** exposure, *c-fos* mRNA expression in the PVN **(A,B,E,F)** and ACC **(C,D,G,H)** were compared between vehicle- and CP-154526-injected pups. ^∗∗^*p* < 0.01. Mean ± SEM.

The PVN showed the high *c-fos* mRNA expression even after CP-154526 pre-injection both in MAB and ISO pups [MAB in **Figures 5A,B**; *t* = -0.73, *df* = 5.89, *p* = 0.50, *n* = 4 (CP-154526), 5 (vehicle); ISO in **Figures 5E,F**; *t* = -1.09, *df* = 5.26, *p* = 0.32, *n* = 4 each group], suggesting the activation of PVN neurons were independent from the CRF-CRFR1 signaling.

Neither the LSI, ACo nor the LPAG showed significant change in *c-fos* mRNA level among vehicle and CP-154526 injected pups after MAB (LSI, *t* = 1.83, *df* = 5.85, *p* = 0.12, *n* = 4 each group; ACo, *t* = 0.64, *df* = 7.77, *p* = 0.54 *n* = 5 each group; LPAG, *t* = 1.26, *df* = 7.99, *p* = 0.24, *n* = 5 each group) or ISO (LSI, *t* = -0.52, *df* = 5.57, *p* = 0.62, *n* = 4 each group; ACo, *t* = 0.62, *df* = 5.37, *p* = 0.56, *n* = 5 each group; LPAG, *t* = -0.49, *df* = 7.8, *p* = 0.64, *n* = 5 each group), consistent to their lack of CRFR1 expression (**Figure 4A**).

*c-fos* mRNA expression in the CeA was hardly detectable either by MAB (mean density (mm^2^) ± SEM is 4.36 ± 0.76 in Vehicle, 5.63 ± 2.86 in CP-154526) or by ISO (5.63 ± 3.48 in Vehicle, 6.25 ± 3.28 in CP-154526), as confirmed in the initial screening of *c-fos* mRNA expression, and with no significant difference among the treatment (MAB, *t* = -1.26, *df* = 2.44, *p* = 0.31, *n* = 3 each group; ISO, *t* = 0.14, *df* = 5.94, *p* = 0.89, *n* = 4 each group).

In contrast, *c-fos* mRNA expressions in the ACC were significantly decreased by CP-154526 pre-injection both in MAB and ISO pups (MAB in **Figures 5C,D**; *t* = -5.99, *df* = 4.03, *p* < 0.01, *n* = 5 each group; ISO in **Figures 5G,H**; *t* = -5.1, *df* = 4.87, *p* < 0.01, *n* = 5 each group), consistent with the effects on the disinhibition of Transport Response (**Figures 3A,C**).

Therefore, among the six areas examined, the CRFR1-expressing ACC neurons were the most plausible candidate to mediate the social context-dependent attenuation of the Transport response. Therefore, we focused our further analyses on the ACC.

### ACC Lesion Prevents Attenuation of Transport Response After Maternal Absence or Social Isolation

**FIGURE 6:**
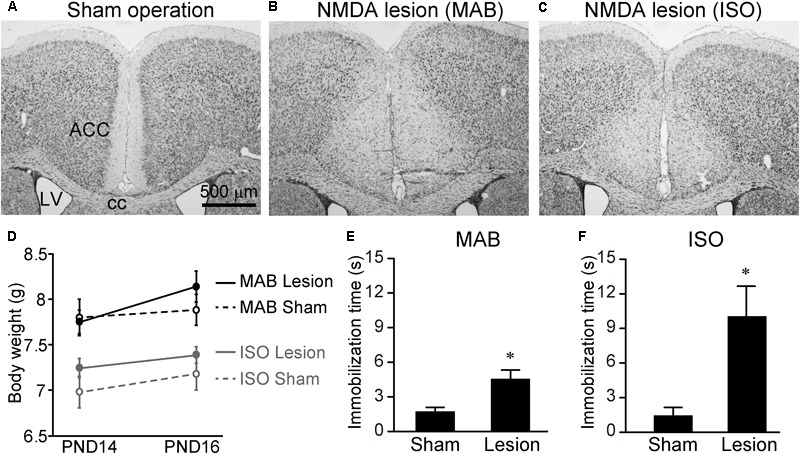
Immobilization time of pups received ACC lesion. PND14 pups received saline or NMDA injection into the ACC. To confirm the lesioned areas, brain sections were stained with thionine **(A–C)**. Body weights were measured at PND14 and PND16 **(D)**. At PND16, immobilization time was compared between sham-operated and NMDA-injected pups after MAB **(E)** or ISO **(F)** exposure. ACC, anterior cingulate cortex; cc, corpus callosum; LV, lateral ventricle. ^∗^*p* < 0.05. Mean ± SEM.

Finally, we examined whether the attenuation of Transport Response by MAB and/or ISO was disinhibited by excitotoxic lesions of the ACC. PND14 pups received NMDA injection into the ACC (**Figures 6A–C**). Consistent with the previous literature (Rangon et al., 2007), there was no significant difference in body weight at PND16 [**Figure 6D**, MAB, *t* = -1.07, *df* = 11.71, *p* = 0.31, *n* = 6 (sham), 8 (lesion); ISO, *t* = -1.03, *df* = 6.11, *p* = 0.34, *n* = 5 (sham), 7 (lesion)] or the general health condition between sham-operated and NMDA-injected pups. At PND16, pups with or without ACC lesions were exposed to either MAB or ISO, and then picked up by experimenters. It turned out that the ACC lesions abolished the social context-dependent regulation of the Transport Response, so that the ACC lesioned pups showed a longer immobilization time than sham-operated pups to the level of the undisturbed pups that stayed with the mother in **Figure 1B** [**Figure 6E**, MAB, *t* = 3.97, *df* = 7.57, *p* < 0.01, *n* = 6 (sham), 8 (lesion); **Figure 6F**, ISO, *t* = 4.25, *df* = 6.14, *p* < 0.01, *n* = 5 (sham), 7 (lesion)]. In addition to body weight, there was no significant difference in the time required righting reflex between NMDA-injected (0.48 ± 0.02 s) and sham-operated (0.47 ± 0.02 s) pups (*t* = 0.52, *df* = 3.98, *p* = 0.63, *n* = 3 for each), suggesting that NMDA-injected pups were not hypoactive.

These results indicated that ACC neurons were not necessary for the Transport Response *per se*, but were responsible for the social-context dependent inhibition of Transport Response.

## Discussion

In this study, we found that the mouse pups attenuate the expression of Transport Response not only after social isolation but also after maternal absence even in their home cage with littermates since PND13. The inhibition of Transport Response under social isolation or maternal absence appears functional, because when a pup is suddenly carried in the air under such conditions, the carrier may not be its mother, but can be a predator. In these cases, struggling seems to be more adaptive than the Transport Response to escape from the predator.

It has been unequivocally shown that ISO induces strong stress responses, including an initial increase (e.g., distress vocalizations) and subsequent decrease of activity, and an elevation of plasma CORT, even during the “stress-hyporesponsive period” in rodent pups (Hofer, 1973; Ackerman et al., 1978; Hofer and Shair, 1978; Hennessy and Ritchey, 1987; Stanton et al., 1988), as reproduced in our results. In contrast, 30 min of maternal absence happens daily to rodent pups, as their mothers regularly leave the nest for foraging in the natural habitat (Grota and Ader, 1974; Hennessy and Sharp, 1990). Still, 30-min of maternal absence does increase the plasma CORT level in PND16 pups and, more surprisingly, inhibits the immobility of Transport Response in pups older than PND13, indicating that a 30-min period of maternal absence is perceived by these pups, and makes them adapt their physiology and behaviors accordingly.

This MAB-induced downregulation of the Transport Response is not observed during PND8–12, and is expressed during PND13–16, suggesting that this behavioral adaptation to MAB requires not only maturation of pups’ motor system but also that of cognitive functions. A similar developmental acquisition of socially mediated behavioral response is observed in “maternal potentiation” of USV of a rat pup (Hofer et al., 1999; Shair, 2007); at around PND10, isolated pups produce USV at a higher rate when the pups experience a brief contact with the mother than they would during an isolation not preceded by maternal contact.

Though MAB caused stress responses including the plasma CORT elevation at least at PND16, the inhibition of Transport Response was not mediated by plasma CORT because, firstly, PND13 pups downregulate their Transport Response without any increase of plasma CORT (**Figure 1A**); and secondly, pups injected with CP-154526 showed longer immobility during the Transport Response under MAB condition, without suppressing the high CORT levels (**Figure 3**). [The CORT elevation observed in CP-154526- or vehicle-injected pups (**Figures 3B,D**) was consistent with the previous literature (Sutton et al., 1982; Eaves et al., 1985; Gozzi et al., 2013); see also Method section for details]. Instead, extra-HPA-axis action of the CRF-CRFR1 signaling seems responsible for the social context-dependent inhibition of Transport Response, because the pharmacological blockade of CRFR1 abolished the downregulation of the Transport Response by MAB and ISO (**Figure 3**). This finding is in concordance with a summary of previous literature (Faturi et al., 2010), suggesting that both short- and long-term effects of maternal separation (in most cases, ISO in our terminology) on stress-related behaviors depends not only on CORT but also heavily on extra-hypothalamic CRF and other neurotransmitter systems.

Several studies have identified the activation of PVN and limbic cortices by isolation ISO (Smith et al., 1997; Horii-Hayashi et al., 2013; Hennessy et al., 2015), but to our knowledge, mapping of acute neuronal activation by pure maternal absence (MAB) has not been performed. We therefore engaged in the brain-wide *c-fos* mRNA expression screening, with a special interest to identify the responsible brain area for the CRFR1-dependent downregulation of the Transport Response under maternal absence. As a result, the activation pattern of the ACC, LSI, ACo, and LPAG matched best with the behavioral outcome during the Transport Response (**Figure 2**). For the hippocampus and CeA, which were implicated in the effects of maternal separation in rats (Caldji et al., 2000; Liu et al., 2000) and were found to be activated by 3 h ISO in mice (Horii-Hayashi et al., 2013), our study failed to detect significant responses either by MAB or ISO. This could be due to the milder level of stress caused by 30 min MAB than by 3 h ISO.

Among the activated areas by MAB, only neurons in the ACC express CRFR1 highly (**Figures 4A–D**) and showed decreased activation by pharmacological blockade of CRFR1 (**Figures 5C,D,G,H**). Moreover, the excitotoxic lesion of the ACC (**Figure 6**) disinhibits the Transport Response under the condition of maternal absence, equal to the CRFR1 blockade. These results indicate that neither the ACC nor CRFR1 is necessary for the Transport Response *per se*, but both are important for sensing maternal absence and modulating the Transport Response accordingly. Considering all these data, it is most prudent to assume that the CRFR1 signaling within the ACC is responsible for the social context-dependent inhibition of Transport Response. The sharp increase of CRFR1 expression level in the ACC observed between PND10 to 16 (**Figures 4E–G**) further supports the time course of maturation in their regulation of Transport Response depending on the social context. The present results do not, however, exclude other possible mechanisms. For example, Sarro and colleagues recorded spontaneous neocortical local field potentials in freely behaving infant rats during natural interactions with their mothers on PND12–19 (Sarro et al., 2014). They showed that the cortical desynchronization of pups in a huddle was highest when the pups were isolated, and higher when the mother was away from the nest yet still within the home cage than when she was in the nest near the pups. Although the maternal absence condition differs between their study and ours, in which the mother is away from the home cage, the common finding is that preweaning rodent pups can sense maternal absence from a nearby environment and respond behaviorally or physiologically. Sarro and colleagues then showed that systemic blockade of noradrenergic beta receptors led to reduced maternal regulation of infant cortical activity. Therefore, both noradrenergic receptor and CRFR1 signaling are involved in the pup’s reaction, and further analyses are required to determine whether these components act in parallel or in a series.

According to the detailed comparative analyses by Vogt and Paxinos (Vogt and Paxinos, 2014), mouse ACC and MCC corresponds to areas 24, 24c, 25, d32, p32, s32, 33 and areas a24′, a24c′, p24′, p24c′, 24d′, 32′, a33′, p33′ in humans, respectively. Caudally to the MCC, the rodent retrosplenial cortex is regarded as human posterior cingulate cortex area 29a–c and 30. Overall, the rodent cingulate cortices possess the topographic homology to those in humans, and the ACC found to be responsible for MAB/ISO-induced suppression of the Transport Response in this study corresponds best to the dorsal part of human ACC such as d32 and 24c.

The function of the ACC remains controversial, but neuroimaging studies in humans showed that ACC plays a major role in cognitive function, emotional processing and decision making (Devinsky et al., 1995; Bush et al., 2000; Rushworth et al., 2007). In particular, the ACC has been shown to be activated by the expectation of negative outcomes related with anxiety and fear (Amit Etkin and Raffael Kalisch, 2011; Euston et al., 2012), such as the distress of their own infants in the maternal ACC (Noriuchi et al., 2008), or disconnection of social bonds (Eisenberger, 2012). In non-human animals, the primate ACC has been repeatedly found to be activated by the subjective experience of pain (Shackman et al., 2011). The rat ACC lesion leads to a similar result in the experience of pain (Johansen et al., 2001), and recent studies suggest a role in behavioral adaptation based on prediction error (Hyman et al., 2017). Considering these previous reports, it is probable that the ACC of mouse pups contributes in sensing maternal absence and inhibiting their attachment behavior accordingly.

In primates and rodents, adult ACC neurons strongly express the CRF receptor CRFR1 (Millan et al., 1986; Potter et al., 1994). It should be noted that the CRFR1 expression level changes dynamically along with postnatal development at many rodent brain areas (Weathington et al., 2014; Zachary et al., 2017), as found in the ACC in this study (**Figures 4E–G**). Low expression of CRFR1 in the PVN has been confirmed throughout the development in the above studies as well as in our study.

For the origin of ligand, CRF, parvocellular neurons in the PVN is the major source of brain CRF. CRF producing neurons are also distributed at the CeA, PAG, and cerebral cortices including the ACC (Swanson et al., 1983; Henckens et al., 2016), raising the possibility that CRF from non-PVN structures or even from the ACC itself acts on CRFR1 in the ACC. While it has been posited that extra-hypothalamic CRF is responsible for stressor-triggered behaviors, a recent study showed that the PVN-specific CRF knockout mice, in which CRF expression was preserved in amygdala and cerebral cortex, showed markedly reduced anxiety behaviors (Zhang et al., 2017). The same study used Crf-cre driver mice and revealed abundant projection from CRF neurons in the PVN to the ACC, which was not identified by the previous study (Swanson et al., 1983), suggesting that the PVN first integrates external information from hypothalamus, brain stem and limbic system, and sends CRF projection to the ACC.

Mouse version of “separation anxiety,” i.e., pups’ total behavioral and physiological changes caused by MAB, should involve not only the CRFR1-dependent pathway that leads to the inhibition of the Transport Response, but also a CRFR1-independent signaling pathway, which activates the PVN neurons and induces CORT elevation (**Figures 3B,D, 5A,B,E,F**). The ACo, LSI, and LPAG, which are activated both by MAB and ISO (**Figure 2**) but do not express CRFR1 (**Figure 4A**) nor reduce their activation by CP-154526 pre-injection, may be involved in such CRF-independent components of separation anxiety. LSI receives massive inputs from hippocampal formation, sends bidirectional projections with the preoptic and hypothalamic areas, and has been consistently found to be involved in stress responses (Risold, 2004; Singewald et al., 2011; Nishi et al., 2013). Afferent and efferent connectivity of the ACo suggests that the ACo may initiate defensive and aggressive responses elicited by olfactory stimuli (Cádiz-Moretti et al., 2017). The LPAG takes a major role in the flight response, receives inputs from the ACC and projects extensively to the rostral ventrolateral medulla that activates preganglionic sympathetic fibers (Bandler and Keay, 1996; Kvetnansky et al., 2009). These CRF-independent pathways may involve noradrenaline and serotonin signaling to exert anxiety-related behavioral and physiological responses; for example, it has been shown that bilateral ibotenic acid lesion of lateral septum increased the HPA axis response and immobility in forced swim test (Singewald et al., 2011). Intraseptal administration of the selective 5-HT1A receptor agonist had the opposite effects, suggesting that the serotonin signaling in the lateral septum is involved in active stress-coping behavior and HPA inhibition.

An interesting recent review paper deals with “maternal buffering,” one kind of social buffering, the phenomenon by which the presence of affiliative social partners mitigates stress responses (Kiyokawa and Hennessy, 2017). And social buffering involves both HPA axis and sympathetic system regulation. Shair and colleagues showed that both a mother and a littermate can reduce the USV of isolated rat pups, but the influence of the mother, not the littermate, is mediated by dopamine receptors (Shair et al., 2009), suggesting that different classes of social partners can induce social buffering via distinct mechanisms. It remains to be clarified, however, whether it is suitable to regard the effects of maternal separation as the lack of “maternal buffering,” particularly in our MAB condition, which does not include any stressor other than the maternal absence *per se*. “Social buffering” implies the attenuation of stress response by social relationship, and may not be appropriate when the condition does not include any other stressor than the lack of social relationship.

Our study showed that the Transport Response in mouse pups becomes sensitive not only to social isolation but also to maternal absence in their home cage with littermate after PND13, at least partly via CRF-CRFR1 signaling in the ACC. The new animal model system provided in this study may lead to understand the neurobiological basis of separation anxiety in mammalian infants.

## Author Contributions

SY designed the experiments, performed them, and wrote the manuscript. RO, RM, and YY-M performed the experiments. YT contributed to establish the lesion study. TK had critically revised the manuscript. HF and KK supervised this study and wrote the manuscript.

## Conflict of Interest Statement

The authors declare that the research was conducted in the absence of any commercial or financial relationships that could be construed as a potential conflict of interest.

## Footnotes

1 http://rsb.info.nih.gov/ij

2 Paxinos and Franklin (2013), Copyright Elsevier.
